# The Injury and Therapy of Reactive Oxygen Species in Intracerebral Hemorrhage Looking at Mitochondria

**DOI:** 10.1155/2016/2592935

**Published:** 2016-05-12

**Authors:** Jie Qu, Weixiang Chen, Rong Hu, Hua Feng

**Affiliations:** Department of Neurosurgery, Southwest Hospital, Third Military Medical University, No. 30, Gaotanyan Street, Chongqing 400038, China

## Abstract

Intracerebral hemorrhage is an emerging major health problem often resulting in death or disability. Reactive oxygen species (ROS) have been identified as one of the major damaging factors in ischemic stroke. However, there is less discussion about ROS in hemorrhage stroke. Metabolic products of hemoglobin, excitatory amino acids, and inflammatory cells are all sources of ROS, and ROS harm the central nervous system through cell death and structural damage, especially disruption of the blood-brain barrier. We have considered the antioxidant system of the CNS itself and the drugs aiming to decrease ROS after ICH, and we find that mitochondria are key players in all of these aspects. Moreover, when the mitochondrial permeability transition pore opens, ROS-induced ROS release, which leads to extensive liberation of ROS and mitochondrial failure, occurs. Therefore, the mitochondrion may be a significant target for elucidating the problem of ROS in ICH; however, additional experimental support is required.

## 1. Introduction

Intracerebral hemorrhage (ICH) accounts for 9–27% of strokes worldwide. It is characterized by poor outcomes, with a high mortality rate of 30–50%, and the neurological outcomes of patients who survive are also very poor [1, 2]. The most common cause of intracerebral hemorrhage is hypertension (in approximately 65% of cases), and many other diseases, including amyloid angiopathy, brain tumours, aneurysms, arteriovenous malformations, cerebral cavernous malformations, and arteriovenous fistulae, also contribute to ICH [3].

Until now, there have been no effective medical or surgical therapies to improve outcomes for ICH patients. Therefore, understanding the manner in which ICH induces brain injury is important in the development of effective treatment. In addition to the initial mechanical injury produced by the hematoma, secondary injuries play an important part in further damage [3]. These secondary injuries include not only nerve cell responses to hematoma-induced stress but also the inflammatory reaction caused by the hematoma and the blood coagulation process. In the pathological process of ICH, brain cells, white matter fibre tracts, and the blood-brain barrier (BBB) are injured by the inflammatory reaction. Reactive oxygen species (ROS) are one of the most important components in the inflammatory reaction because they are both products of and participants in the reaction, causing a vicious circle.

Reactive oxygen species (ROS) are created as part of normal cellular metabolism and defence systems. Under physiological conditions, there is a balance between ROS and the antioxidant system; therefore, ROS are regulated by the antioxidant system and kept at a low level. They can take part in many cellular pathways by modulating a number of kinases, phosphatases, redox-sensitive transcription factors, and genes, which contribute to the regulation of cellular growth, differentiation, proliferation, and apoptosis [4]. However, during ICH, there are additional sources of ROS. Greater amounts of ROS can break the dynamic balance between the antioxidant system and ROS, causing cellular injury in the form of lipid peroxidation, DNA damage, and protein oxidation [5]. Therefore, ROS play an important part in the pathophysiology of ICH. ROS can initiate apoptosis and disrupt the blood-brain barrier (BBB), producing damage to the brain [6, 7]. In this review, we will cover current research to understand ICH-related ROS, including their sources, their injurious effects, their molecular mechanisms, and their relation to the antioxidant system. In addition, we will also summarize therapeutic antioxidant agents and some problems, which may contribute to the development of new therapeutic approaches.

## 2. Reactive Oxygen Species

Reactive oxygen species are highly reactive and short-lived molecules, including free radicals, such as the superoxide anion radical (O_2_^•^) and the hydroxyl radical (^•^OH), and nonradical oxidants, such as hydrogen peroxide (H_2_O_2_) and singlet oxygen (^1^O_2_) [8]. The initial step for ROS production is the univalent reduction of molecular oxygen (O_2_) to form superoxide O_2_^•^. Under normal physiological conditions, this process is mostly mediated by the mitochondrial electron transport chain (ECT) [9]. Electrons can leak from complexes I and III and are free to react with O_2_ to form the superoxide O_2_^•^ that is then catalysed by superoxide dismutase (SOD) to form hydrogen peroxide (H_2_O_2_), which can be processed into the hydroxyl radical (^•^OH) [10]. The ECT, NADPH oxidase, monoaminoxidase, p66^shc^, *α*-glycerophosphate dehydrogenase, electron transfer flavoprotein (ETF) and ETF dehydrogenase, and aconitase may also contribute to the production of ROS in mitochondria [8]. Normally, most ROS can be neutralized by the antioxidant system to maintain cellular homeostasis. For example, catalase and glutathione peroxidase can convert hydrogen peroxide (H_2_O_2_) to water. The “residual ROS” are also used as second messengers. They can take part in many cellular processes, such as proliferation and survival; ROS homeostasis and antioxidant gene regulation; mitochondrial oxidative stress, apoptosis, and ageing; iron homeostasis through iron-sulfur cluster proteins; and the ATM-regulated DNA damage response [4]. However, once the balance between ROS and the antioxidant system is broken by some sudden attack, such as ICH, the antioxidant system cannot eliminate the excess ROS, leading to ROS accumulation in the tissue environment. This will damage the mitochondria and lead to additional ROS release, triggering a cascade of damage in the cell.

## 3. The Sources of ROS in ICH

### 3.1. Hemoglobin Metabolic Products

**Figure 1:**
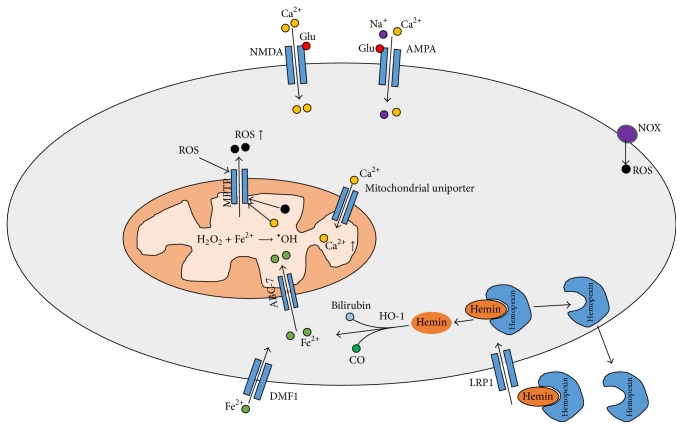
The sources of ROS in ICH: the MPTP can be opened by ROS and Ca^2+^, followed by ROS release. NMDA receptor activation by glutamate causes cellular Ca^2+^ overload, whereas APMA receptors also contribute to Ca^2+^ overload in the mitochondria. Ferrous iron can be transported into the cell through DMF1 and consequently loaded into the mitochondria by ABC-7; hemin binds with hemopexin and is transported into the cell through LRP1; then, inside the cell, hemin is catalysed by HO-1 into ferrous iron, which is then transported into the mitochondria; ferrous iron is used in the reaction to transform H_2_O_2_ into the hydroxyl radical, which is a very active radical in oxidative damage. In addition, ROS can also be produced by NOX (ROS: reactive oxygen species; Fe^2+^: ferrous iron; AMPA: *α*-amino-3-hydroxy-5-methyl-4-isoxazole-propionic acid receptor; O_2_^•^: superoxide radical; ^•^OH: hydroxyl radical; IRP-2: iron regulatory protein-2; NMDA: N-methyl-D-aspartic acid receptor; fer-1: ferrostatin-1; DMT1: divalent metal transporter 1; HO-1: hemoxygenase-1; MPTP: mitochondrial permeability transition pore; NADPH: adenine dinucleotide phosphate; NOX: adenine dinucleotide phosphate oxidase).

After intracerebral hemorrhage (ICH), hematoma and perihematomal regions are rich with RBC lysis products, especially hemin (Figure 1). After intracerebral hemorrhage, red blood cells (RBC), which are present in the hematoma, lyse and release hemoglobin (Hb), which will be degraded to hemin. Hemin can be bound by hemopexin in the serum, and then the complex is transported into the cell via lipoprotein receptor-related protein (LRP1) [11]. Intracellular hemin is degraded into Fe^2+^, bilirubin, and carbon monoxide (CO). Fe^2+^ derived from hemin can generate a hydroxyl radical, which is the most reactive of all oxygen radicals, via the Fenton reaction, leading to oxidative stress [11, 12]. Hydrocephalus after ICH is also related to iron accumulation [13]. Oxidative stress is very obvious on days 1 and 3 after ICH, but mice pretreated with deferoxamine (DFX) exhibited decreased iron accumulation and neuronal death, attenuated production of reactive oxygen species, reduced microglial activation without affecting astrocytes or neutrophil infiltration, and attenuated white matter damage [14]. Iron regulatory protein-2 (IRP2) showed effects on ferritin expression and then affected iron metabolism and neuronal vulnerability to hemoglobin. Reactive oxygen species formation and heamoxygenase-1 expression after hemoglobin treatment were also attenuated by deletion of the IRP2 gene. These results suggest that IRP2-binding activity increases the vulnerability of neurons to hemoglobin, possibly by reducing ferritin expression [14, 15]. In addition to iron and hemoglobin, bilirubin oxidation products (BOXes) may contribute to ROS release in ICH. Clark et al. reported that production of BOXes via the hemoglobin/Fenton reaction under in vivo conditions has been seen following ICH. In hematomas from a porcine ICH model, the authors observed significant production of BOXes, malondialdehyde, and superoxide dismutase, indicating a potent oxidizing environment [16].

### 3.2. Excitatory Amino Acids

The initial bleed leads to an influx of glutamate from the bloodstream, and glutamate is one of the most important damaging factors in nervous system, inducing Ca^2+^ overload, which can lead to membrane depolarization and ROS release. Neurons are highly vulnerable to glutamate-induced excitotoxicity. Some evidence shows that glutamate can also participate in brain injury after intracerebral hemorrhage [15]. Activation of the NMDA receptor by glutamate increases Ca^2+^ influx, which mediates an excessive rise in cytosolic Ca^2+^ and consequent mitochondrial Ca^2+^ loading. In addition, during the pathophysiologic process of ICH, production of thrombin after hemorrhage results in activation of Src kinase, which phosphorylates NMDA receptors, enhancing their function [17]. In addition, activation of AMPA receptors also contributes to the influx of Ca^2+^ and Na^+^, which leads to mitochondrial Ca^2+^ loading, and this process can be blocked by ruthenium red, a mitochondrial calcium uniporter blocker [18]. Mitochondrial Ca^2+^ loading contributes to the decrease in transmembrane potential and the opening of the mitochondrial permeability transition pore (MPTP), causing damage to the mitochondria and mitochondrial respiratory chain and consequent ROS release [19].

### 3.3. Inflammatory Cells

Microglial activation also contributes to the pathogenesis of brain injury in intracerebral hemorrhage (ICH). Tsirka has reported that inhibiting microglial activation and macrophage infiltration by the tripeptide macrophage/microglial inhibitory factor (MIF) Thr-Lys-Pro attenuated the numbers of ethidium-positive cells compared with saline-treated control mice, reduced production of reactive oxygen species, and improved neurological functional outcomes [20]. In addition, granulocytes can also be a source of ROS after ICH. They can cause the release of ROS via NADPH oxidase and myeloperoxidase [21]. Although these processes are necessary for antimicrobial defence, high ROS levels due to microglial activation and neutrophil infiltration contribute to poor outcomes after ICH [6, 22].

### 3.4. ROS-Induced ROS Release

The mitochondrial permeability transition pore (MPTP) is a multiprotein complex comprising cyclophilin D, a mitochondrial peptidyl-prolyl cis-trans isomerase; voltage-dependent anion channel (VDAC); adenine nucleotide translocator (ANT); and other molecule(s) that forms a channel in the mitochondrial inner membrane [23]. Its opening plays an important physiological role in maintaining healthy mitochondrial homeostasis (Figure 1). Adaptive and maladaptive responses to redox stress may involve mitochondrial channels such as the mPTP and the inner membrane anion channel (IMAC). The activation of these channels causes intracellular and intramitochondrial redox-environment changes, leading to ROS release. This regenerative cycle of mitochondrial ROS formation and release is named ROS-induced ROS release (RIRR) [8]. At higher levels of ROS, longer mPTP openings may release an ROS burst, leading to destruction of the mitochondrion and, if propagated from mitochondrion to mitochondrion, of the cell itself. Therefore, mitochondria are an important source of ROS. Following ICH, the mPTP was formed, and mROS increased, but these effects could be reversed by the VDAC inhibitor, TRO-19622, or the mROS-specific scavenger, Mito-TEMPO [24].

## 4. The Contribution of ROS to Brain Injury

### 4.1. Brain Cell Injury

**Figure 2:**
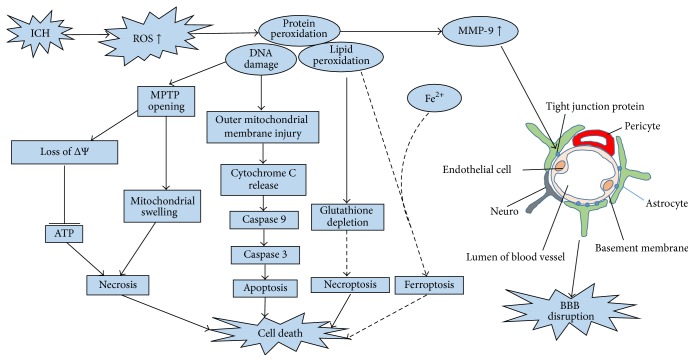
Many additional reactive oxidative species are produced after ICH. They can cause cell death and BBB disruption in the form of lipid peroxidation, DNA damage, and protein peroxidation, which contribute to the opening of the MPTP and to injury of the outer mitochondrial membrane. Once the MPTP opens, many molecules will enter into the mitochondria, and the ΔΨ will decrease, injuring the mitochondrial respiratory chain and causing ATP depletion, causing mitochondrial swelling and necrosis. In addition, ROS can injure the outer mitochondrial membrane, causing cytochrome c release and activating caspase-dependent apoptosis. Excessive ROS can also cause glutathione depletion, leading to necroptosis. Ferroptosis, characterized by iron-dependent accumulation of ROS, may be a potential apoptotic mechanism during ICH. ROS also can upregulate the expression of MMP-9, degrading tight junction proteins and the basal laminar proteins, leading to BBB disruption (ICH: intracerebral hemorrhage; ROS: reactive oxygen species; BBB: blood-brain barrier; MPTP: mitochondrial permeability transition pore; ΔΨ: transmembrane potential; MMP-9: matrix metallopeptidase 9).

ROS can cause cellular injury in the form of lipid peroxidation, DNA damage, and protein peroxidation, but organisms also can utilize a series of antioxidant defences, which will be discussed in next section, to protect against oxidative damage [5] (Figure 2). However, ICH-induced ROS are so abundant that antioxidant defences cannot neutralize them, leading to apoptosis through several mechanisms. The classical mechanism is caspase-dependent death: oxidative stress caused by ROS has been shown to induce cytochrome c release, which is often the initiation of apoptosis [6]. ROS can permeabilize the outer mitochondrial membrane, mobilizing cytochrome c from the intermembrane space into the cytosol. Released cytochrome c causes formation of the apoptosome and activates initiator caspase 9, which then activates caspase 3, leading to the inevitable onset of apoptotic death [25]. There may be other apoptotic mechanisms in addition to caspase-dependent death. In a simplified in vitro model of hemoglobin neurotoxicity, upstream and downstream caspases were upregulated, but caspase inhibition did not result in neuroprotection, and a free radical scavenger significantly reduced neuronal death, which indicated that another parallel pathway related to oxidative stress may contribute to cell death [26]. Ferroptosis, a newly recognized form of programmed cell death characterized by iron-dependent accumulation of ROS, similar to the pathological process in ICH, may be a potential apoptosis mechanism in ICH [27]. This type of pathway is found in tumour cells, and expression of ferroptosis-related genes, such as lipocalin-2 (LCN2), a protein that participates in iron homeostasis and enhances brain iron clearance after ICH, can be tested in ICH [15]. However, existing evidence for ferroptosis is nonspecific, and more specific biomarkers are needed to characterize the pathway in ICH. Necroptosis is a type of programmed cell death that has been found in the pathological process of ICH [28, 29]. It is characterized by defined molecular mechanisms and can be inhibited by necrostatin-1. Reactive oxygen species are also involved in the regulation of necroptosis, and glutathione depletion may play a role in necroptotic astrocyte injury after ICH [30]. The current minireview discusses the evidence for and against a role for reactive oxygen species in necroptosis. In addition, activation of the mPTP, a pore channel in the mitochondrial membrane, may also be a potential mechanism for necrosis and apoptosis. ROS produced during ICH can attack the mPTP. Once the mPTP opens, it will allow water, large molecules, and iron ions to enter into the mitochondrial matrix, leading to impairment of the mitochondrial respiratory chain (MRC), which results in a greater release of mitochondrial reactive oxygen species (mROS) and cell death [8]. This process has been demonstrated in ischemic stroke, but there is little research about MPTP in ICH. In an ICH mouse model, Ma et al. proved that, following ICH, MPTP is activated, causing an increase in mROS; however, the activator of the MPTP was not mentioned. Therefore, more evidence is needed to demonstrate that the MPTP can be activated by ROS after ICH.

### 4.2. Injury to the BBB

The blood-brain barrier (BBB) is a dynamic interface between the peripheral circulation and the central nervous system (CNS) that prevents toxic substances from entering into the CNS and contributes to the maintenance of brain homeostasis [31]. The BBB is formed by capillary endothelial cells, which are connected by tight junctions (TJ), together with closely associated astrocytes, pericytes, and neurons, as well as the extracellular matrix [31]. Disruption of the BBB can cause brain edema, which is an important secondary injury after ICH. Edema is related to oxidative stress (Figure 2). Matrix metalloproteinases (MMPS) comprise a family of zinc-endopeptidases that can degrade TJ proteins and basal laminar proteins, increasing the permeability of the BBB. Many researchers have demonstrated that MMPs, particularly MMP-9, are upregulated after ICH, which is associated with oxidative stress [32]. Inhibition of oxidative stress via SOD1 overexpression also can decrease MMP-9 levels [7]. Therefore, ROS can trigger numerous molecular cascades that mediate the activation of MMPs, leading to BBB disruption.

## 5. Antioxidant System in Intracerebral Hemorrhage

**Figure 3:**
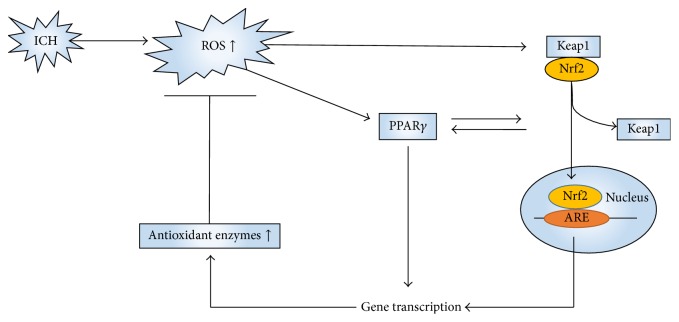
Oxidative stress can activate pathways involving Keap1 and Nrf2. Keap1 is a detector of ROS and a negative regulator of Nrf2. Under physiological conditions, Nrf2 is in a dormant state. When the brain is exposed to oxidative stress caused by ICH, Nrf2 will dissociate from Keap1, translocate to the nucleus, and activate antioxidant response element- (ARE-) dependent gene expression to neutralize ROS. Activation of PPAR*γ* can lead to upregulation of the antioxidant enzymes catalase and superoxide dismutase (SOD). Nrf2: nuclear factor erythroid 2-related factor; Keap1: Kelch-like ECH-associated protein 1; ICH: intracerebral hemorrhage; ROS: reactive oxygen species; PPAR*γ*: peroxisome proliferator-activated receptor gamma.

To neutralize the rising ROS formed in ICH, the cellular antioxidant system is activated. The pathway involving Kelch-like ECH-associated protein 1 (Keap1) and nuclear factor erythroid 2-related factor 2 (Nrf2) is known as the major endogenous antioxidant system [33]. It was first discovered in studies of anticarcinogenic compounds, and a beneficial role of the pathway in the progression of ICH has been demonstrated [34] (Figure 3). Nrf2 knockout (Nrf2^−/−^) mice have greater ROS production and DNA damage than wild type (WT) mice when subjected to 24 h of ICH [35]. Keap1 is a detector of ROS and a negative regulator of Nrf2. Under physiological conditions, Nrf2 is anchored within the cytoplasm by Keap1. When the brain is exposed to oxidative stress caused by ICH, Nrf2 will dissociate from Keap1, translocate to the nucleus, and activate antioxidant response element- (ARE-) dependent gene expression [35]. Shang et al. reported that, after blood infusion in a rat model, the expression of Keap1 began to decrease at 8 h, whereas Nrf2 began to show a significant increase at 2 h, with a peak at 24 h [36]. As a result of ARE's activation, different antioxidant enzymes, including superoxide dismutase (SOD), glutathione (GSH), hemeoxygenase-1 (HO-1), glutathione-S-transferase (GST), catalase, NADPH quinone oxidoreductase-1 (NQO1), and thioredoxin (TRX), are upregulated [35]. These enzymes can partly protect the cell from the oxidative stress caused by ICH. This pathway has also become a key target for therapies in ICH because of its pleiotropic antioxidant effects. Sulforaphane, an agent that can upregulate Nrf2, reduced injury caused by ICH in mice and rats via an Nrf-dependent mechanism [37]. Chang et al. found that, in the ICH mouse model induced by injecting collagenase, (−)-epicatechin, a brain-permeable flavanol, reduced the volume of the lesion and ameliorated neurologic deficits via activation of Nrf2-dependent and Nrf2-independent pathways [38]. Sukumari-Ramesh and Alleyne reported that tert-butylhydroquinone, a selective inducer of Nrf2, attenuated neurodegeneration and improved neurological outcomes after ICH [39]. Furthermore, peroxisome proliferator-activated receptor gamma (PPAR*γ*) (Figure 3), which has pleiotropic effects regulating anti-inflammation and playing a role in glucose and lipid metabolism, appears to regulate the expression of Nrf2 [40, 41]. Activation of PPAR*γ* also may directly lead to upregulation of antioxidant enzymes, such as catalase and superoxide dismutase [42, 43]. Therefore, PPAR*γ* may be another endogenous antioxidant system.

## 6. Clinical Significance of ROS in ICH

### 6.1. Biomarkers in ICH

To understand the pathophysiology of oxidative stress in intracerebral hemorrhage and to identify patient outcomes, we should take measures to evaluate oxidative stress. However, direct measurement of ROS in the brain is still difficult in humans. Therefore, several biological substances relevant to oxidative stress have been investigated as potential peripheral markers. In ischemic stroke, much research related to these biomarkers has demonstrated that biomarkers can be divided into two groups: (1) biomolecules damaged by ROS, including malondialdehyde (MDA); oxidized low-density lipoproteins (oxLDL); 8-isoprostaglandin-F-2 (8-iso-PGF2), a biomarker of lipid peroxidation; and 8-hydroxy-2-deoxy-guanosine (8-OHdG), a biomarker of DNA oxidation; (2) enzymes and molecules related to the antioxidant defence system, including superoxide dismutase (SOD), glutathione peroxidase (GPX), thioredoxin (Trx), and gamma-glutamyltransferase (GGT); vitamins A (retinol), C (ascorbic acid, AA), and E; and carotenoids [44]. However, during the pathological process of ICH, not all of the biomarkers mentioned in this passage can be detected. In a clinical study including 178 individuals (64 patients, 114 controls), ICH was significantly associated with an increased level of 8-OHdG, decreased GPX activity, and a decreased level of vitamin E, whereas MDA and vitamin A levels were not associated with ICH risk, and leukocyte 8-OhdG was better than traditional factors in predicting ICH outcome [45]. Uric acid (UA), an antioxidant molecule in human plasma, provides effective protection against oxidative stress in models of stroke. However, the relationship between uric acid and ICH prognosis is controversial; some authors have found that high levels of UA were correlated with a poor prognosis in ICH [46], whereas others did not conclude that uric acid levels were correlated with outcomes in ICH patients [47]. Vitamin C (ascorbic acid, AA) levels were also important indicators in ICH patients. AA levels were significantly inversely correlated with the severity of neurological impairment, as assessed by the Glasgow Coma Scale and the National Institutes of Health Stroke Scale, and with the major diameter of the lesion [48]. In conclusion, biomarkers of oxidative stress in ICH are similar to those of ischemic stroke, but there are differences between ICH and ischemic stroke, and assessment of multiple biomarkers may provide a better view of oxidative stress and outcomes in ICH patients.

### 6.2. Antioxidant Therapy in ICH

Therapeutic agents can be divided into two groups. One group includes agents that can prevent the formation of free radicals. Iron is a reactant in the formation of the hydroxyl radical, a highly reactive ROS, via the Fenton reaction, and iron chelating agents, such as clioquinol (CQ) and deferoxamine, reduced ICH-induced brain edema, neuronal death, and brain atrophy in a rat ICH model [49–51]. Apocynin, an inhibitor of NAD(P)H oxidase, can delay cerebral vasospasm in a rat subarachnoid hemorrhage model [52], and it also has protective effects on pups with intraventricular hemorrhage [53]. However, a neuroprotective effect was not observed in a rat ICH model [54]. Perhaps the amount of ROS derived from NAD(P)H oxidase is small compared with the total amount of ROS produced in ICH such that the effect of apocynin is limited. Sulfaphenazole (SPZ), which can inhibit superoxide production by cytochrome P450, is also a ROS scavenger. Hama et al. reported that systemic SPZ treatment reduces striatal dysfunction, elevated lipid peroxidation, and brain edema in a rat ICH model [55]. Thrombin can also initiate potentially harmful pathways, such as apoptosis in cultured neurons and astrocytes [56], and can activate Src kinase [57, 58], which may contribute to excitotoxicity and ROS release [59]. Therefore inhibition of thrombin may reduce injury induced by intracerebral hemorrhage. Mitochondria play an important role in ROS release in ICH, as mentioned in Section 3. Ma et al. reported that the mitochondrial ROS scavenger Mito-TEMPO can decrease the amount of the ROS in ICH and that TRO-19622, an inhibitor of the MPTP, can also reduce RIRR [24].

Agents that scavenge free radicals constitute the second group. These compounds include melatonin (5-methoxy-*N*-acetyl-tryptamine) and its metabolites, which are able to ameliorate early brain injury [60–62]. Melatonin has been shown to inhibit ROS related to red blood cell lysis and hemoglobin degradation. In addition, it can increase the expression of Nrf2-ARE pathway-related free radical scavengers, such as SOD and GSH, by activating the Nrf2-ARE pathway [63]. Hydrogen, the lightest element in the periodic table and the most abundant chemical substance in the universe, also can be used as an effective antioxidant therapy. Ohsawa et al. reported that inhaled hydrogen gas has antioxidant and antiapoptotic properties that protect the brain against ischemia-reperfusion (I/R) injury and stroke by selectively reducing the hydroxyl radical [64]. In the ICH model, inhalation of hydrogen gas can reduce brain edema acutely (24 h), but it is not effective over a longer time frame (72 h) [65].* Momordica charantia* polysaccharide (MCP), obtained from* Momordica charantia*, can also be an antioxidant agent. Duan et al. also have demonstrated that MCP scavenged ROS in intracerebral hemorrhage damage, attenuating neuronal death in primary hippocampal neurons [66]. Pyrroloquinoline quinone (PQQ), which has been proven to exist in various fruits, vegetables, milk, and even mammalian tissues, can antagonize oxidative stress-induced cell damage [67–69]. Lu et al. have reported that PQQ reduced the production of reactive oxygen species, alleviated brain edema, and improved locomotor function after ICH [70]. Redox nanoparticles may become a new type of treatment for ROS. These agents can solve the problems of traditional agents, including nonspecific dispersion in normal issues, preferential renal clearance, poor permeability across the BBB, and rapid reduction to the corresponding hydroxylamine form. Chonpathompikunlert et al. reported that, in rats treated with redox polymer self-assembled nanoparticles (nitroxide radical-containing nanoparticles [RNPs]), significantly lower levels of superoxide anion and 8-OHdG (biomarkers of oxidative stress) were detected compared with rats in the control group. RNPs also ameliorated intracerebral hemorrhage-induced brain edema and neurological deficits [71].

There are many studies on antioxidant strategies in ICH, but there have been only a few drugs tested in clinical trials. Edaravone, a free radical scavenger, can significantly improve the outcome of ischemic stroke, as evaluated by the modified Rankin Scale, within 3 months and was introduced in Japan for clinical use in June 2001 [72]. Its neuroprotective effect was also proven in preclinical trials [73–75]. However, in a meta-analysis of edaravone for acute intracerebral hemorrhage, which included 10 randomized controlled trials (RCTs) involving 768 participants, although edaravone treatment increased the rate of improvement of neurological impairment within the scheduled treatment, it is not clear that this translated to any longer-term benefit of clinical importance, and the quality of each trial was not high [76]. Therefore, the longer-term benefit of edaravone is still unclear. NXY059, a free radical-trapping neuroprotectant, is also a clinically tested antioxidant. However, in a randomized control trial including 607 patients, there were no differences in 3-month function, disability, or neurological deficit scores between the experimental group and the control group [77].

## 7. Conclusions

Reactive oxygen species are increasingly recognized as important players in the pathophysiology of secondary brain damage after ICH. Mitochondria play an important part in the production of ROS and are necessary to the processes of neuronal cell death and BBB injury. Much effort has been put into the development of antioxidants to neutralize oxidative stress. We have investigated many intracellular and mitochondrial targets, aiming to decrease ROS release or eliminate released ROS in ICH. However, we know that nearly all attempts failed. There may be two reasons for this. ROS in cells are not always harmful; they also play a role in molecular signal transduction. Therefore, treatment should be aimed at cytotoxic radicals. Additionally, we should pay more attention to the time window in which drugs are administered because it is not easy to supply patients with drugs in time. Moreover, the local and systemic influences of reactive oxygen species in ICH patients remain to be better characterized.
